# Time Reproduction and Numerosity Interaction in the Parietal Cortex: Some Missing Links

**DOI:** 10.3389/fneur.2013.00045

**Published:** 2013-05-06

**Authors:** Carmelo M. Vicario

**Affiliations:** ^1^School of Psychology, The University of QueenslandSt Lucia, Brisbane, QLD, Australia; ^2^Faculty of Motor Science, University of PalermoPalermo, Italy

The interaction of time and numerical representation in the human brain is currently object of debate in the field of cognitive neuroscience. A wide amount of research based on “A theory of Magnitude (ATOM)” (Walsh, 2003), a model which proposes common cortical metric for the representation of space, time, and numbers, has provided extensive evidence in support of this suggestion (Dormal et al., 2006; Xuan et al., 2007; Oliveri et al., 2008; Vicario et al., 2008, 2012; Lu et al., 2009; Vicario, 2011). For instance, it has been shown that merely looking at numbers causes a bias in a time-bisection task that depends on its magnitude (Vicario, 2011). Nevertheless, there are also works which contrast with such proposal as it seems that these two dimensions refer to at least partially separated neural segregations (for instance, see Dormal et al., 2008; Agrillo et al., 2010). Thus, we are only beginning to understand the complex neuronal mechanisms underlying the interaction of these magnitudes in the human brain.

In a recent issue of the *Journal of Neuroscience*, Hayashi et al. (2013) added new insight into the current debate on the interaction of time and numerosity by exploring their neural correlates. The use of transcranial magnetic stimulation (TMS) for the investigation of this issue in the human brain is not new in the literature, as an attempt was previously made by Dormal et al. (2008). However, in the current work Hayashi et al. (2013) have used a clever methodology for testing their hypothesis as they applied TMS upon the neural regions which resulted in a joint activation during the execution of time and quantity processing tasks. Moreover, they used two different timing tasks (time discrimination vs. time reproduction) with the purpose of exploring the time numerosity interaction in the presence and in the context of a reduced involvement of categorical magnitude judgment. Remarkably, this work shows that TMS upon the right inferior frontal gyrus (IFG) impairs categorical duration discrimination, but in contrast, it has no effect on time estimation in the duration reproduction task. On the other hand, the TMS upon the right intraparietal cortex (IPC) modulates the degree of influence of numerosity on time reproduction and impairs precise time estimation. This evidence is striking insofar as it provides clear evidence of a common neural origin for the processing of time and numerosity in a precise region of the parietal cortex. The provided results lead the authors to propose a two-stage model of numerosity–time interactions, in which it is stated that the categorical decision takes place in the frontal cortex, whereas the interaction of numerosity information on perception of time occurs within the parietal cortex. Nevertheless, in the context of the present discussion there are two aspects worthy of receiving attention: the “*motor*” nature of the time reproduction task and the involvement of hand/fingers body part for its execution.

Previous studies have shown neural activity in the parietal cortex for particular visuomotor actions involving right hand/fingers representation such as grasping (Baumann et al., 2009), and the go/no go task (Sugawara et al., 2013). Interestingly, this parietal activation occurred after the Go stimulus, which can be considered the equivalent of the stimulus disappearance in the time reproduction task, of Hayashi et al. (2013). In fact, in this task participants were instructed to start the time reproduction action once the stimulus (digit or dot arrays) disappeared from the computer screen. It is also interesting to note that the parietal cortex of the right hemisphere is directly involved in motor tasks performed with the right hand. For instance, Hinkley et al. (2009) have recently reported that two regions of the right posterior parietal cortex were active in all the experimental subjects asked to perform a grasping task with their right hand. Thus, in consideration of this evidence, it is not possible to exclude that TMS upon the right IPC might affect performance by modulating the accuracy with which participants performed the required movement with their right hand, rather than their timing ability. In fact, by using a time reproduction it is not possible to disentangle the effect played by TMS on the motor and temporal dimensions of this task.

In strict connection with this argument is the close relationship between the representation of numerosity and hand/fingers in the parietal cortex, as showed by the common parietal activation for finger movements (Sugawara et al., 2013) and numerical cognition (Dormal et al., 2008). Moreover, previous works have extensively shown that, in both forced (Dehaene et al., 1990) and free-response (Daar and Pratt, 2008; Vicario, 2012) paradigms, perceiving numbers affects the execution of fingers action. The relationship between fingers and numerosity is also supported by the study of Costa et al. (2011) showing that deficits in fingers gnosia were found in association to mathematical difficulties. Interestingly, the authors provide argument that the deficits in fingers gnosia could not be attributed to a shortage in working memory capacity but rather to a specific inability to use fingers to transiently represent magnitudes. All these works provide further support to the argument of a close relationship between hand/fingers and numerical magnitude through different experimental designs. Although the studies above mentioned have explored mechanisms qualitatively different from those investigated by Hayashi et al. (2013), the involvement of the hand/fingers at motor and/or representational level represents the *trait d’union* among all of these studies.

Taken together these results suggest that there may be a limit related with the use of the time reproduction task for testing the interaction of time and numerosity since it becomes difficult to establish: (i) if numerosity modulates performance by affecting the execution of the hand/fingers movements or the temporal representation of participants; (ii) if the effect played by TMS on the current timing task is due to its modulatory effect on hand/fingers motor planning rather than on timing performance. To overcome these concerns a good control would have been to subject participants to a non-timing motor task which involves the hand also (as in the case of the current time reproduction task), to ensure that the reported effect is not related to the execution of the action and/or to the hand/fingers involvement. Perspective works devoted to dissociate the interaction between time and numerosity might focus on experimental paradigms that are able to control the factors mentioned above (action and hand/fingers representation). The use of a *verbal time estimation* task (Hurks and Hendriksem, 2011) could represent a valid solution to clear these limits. In fact, this task does not require the involvement of hand/fingers movements.
